# Idiopathic splenic vein stenosis with splenic infarction: a case report of rare non-bleeding cause of left-sided portal hypertension

**DOI:** 10.1186/s12876-026-04783-9

**Published:** 2026-04-10

**Authors:** Seth Kofi Abrokwa, Johannes Lenz, Leonore Serfling, Claudia Bielecke, Mathias Schumann, Michael Zaenker

**Affiliations:** 1 Department of Internal Medicine, Immanuel Hospital Bernau, Heart Center Brandenburg, Bernau bei Berlin, Germany; 2 University Hospital of the Medical School Brandenburg Theodor Fontane, Neurupin, Germany; 3 Poznan University of Medical Sciences, Poznan, Poland; 4 Radiology Practice, Immanuel Hospital Bernau, Bernau bei Berlin, Germany

**Keywords:** Idiopathic splenic vein stenosis, Gastric varices, Splenic infarction, Portal vein thrombosis, Cavernous transformation, Splenectomy

## Abstract

**Background:**

Idiopathic splenic vein stenosis is a rare cause of left-sided portal hypertension, typically presenting with gastric varices and bleeding risk. Non-hemorrhagic presentations including those associated with splenic infarction, have not been reported.

**Case presentation:**

A 71-year-old woman presented with persistent epigastric discomfort. Endoscopy revealed type I isolated gastric fundal varices, and contrast-enhanced Computer tomography confirmed high-grade splenic vein stenosis, collateral vessels, a wedge-shaped splenic infarction, moderate splenomegaly, and incidental celiac trunk and superior mesenteric artery stenoses. Laparoscopic splenectomy was performed due to symptomatic infarction and bleeding risk, resolving discomfort. Postoperative follow-up revealed portal vein thrombosis with cavernous transformation, managed with anticoagulation. Gastroscopic follow-ups post-splenectomy showed near-complete variceal resolution and no esophageal varices.

**Conclusion:**

This case highlights a rare non-bleeding presentation of idiopathic splenic vein stenosis with splenic infarction, the diagnostic utility of advanced imaging, and the role of splenectomy for symptom and variceal resolution. Postoperative portal vein thrombosis suggests a possible underlying vasculopathy, warranting vigilant surveillance and further study.

**Supplementary Information:**

The online version contains supplementary material available at 10.1186/s12876-026-04783-9.

## Introduction

Splenic vein stenosis (SVS) is a rare cause of left-sided portal hypertension, characterized by splenic vein narrowing leading to isolated gastric fundal varices, splenomegaly, and occasionally hypersplenism, with normal liver function [1–4]. Unlike secondary SVS, often caused by pancreatic pathology, idiopathic SVS, lacking identifiable etiology is exceptionally rare and poorly understood [5]. Hypotheses for its pathogenesis include congenital venous anomalies or idiopathic fibrosis [6, 7]. The typical presenting complains is hematemesis although patients may present with nonspecific symptoms like epigastric discomfort, complicating diagnosis. Multiphasic contrast-enhanced Computer Tomography (CT) or Magnetic resonance imaging (MRI) is essential for assessing splenic vein patency and collateral formation [8]. Laparoscopic splenectomy is the standard treatment for symptomatic cases, effectively relieving symptoms and decompressing varices [9, 10]. Post-splenectomy portal vein thrombosis (PVT) is a recognized risk in splanchnic surgeries, necessitating vigilant surveillance [11], but has not been reported in relation to idiopathic SVS. We report a unique case of idiopathic SVS with splenic infarction and postoperative PVT, highlighting diagnostic and management challenges.

## Case presentation

### Clinical presentation

A 71-year-old woman with no significant past medical history presented to her general practitioner with insidious-onset, non-radiating epigastric discomfort persisting for several weeks unresponsive to empirical proton pump inhibitor therapy. She denied gastrointestinal bleeding, constitutional symptoms or changes in bowel habits. Given the persistence of symptoms and absence of a clear etiology, she was referred to our internal medicine outpatient clinic for further evaluation. Physical examination was unremarkable. Laboratory investigations showed a normal complete blood count and liver function profile, except for mild thrombocytopenia as shown in Table 1, which documents key hematological, biochemical, rheumatological findings in the supplement material.

Upper gastrointestinal endoscopy was subsequently scheduled to investigate possible gastric or esophageal causes of her symptoms.

### Diagnostic findings

**Fig. 1:**
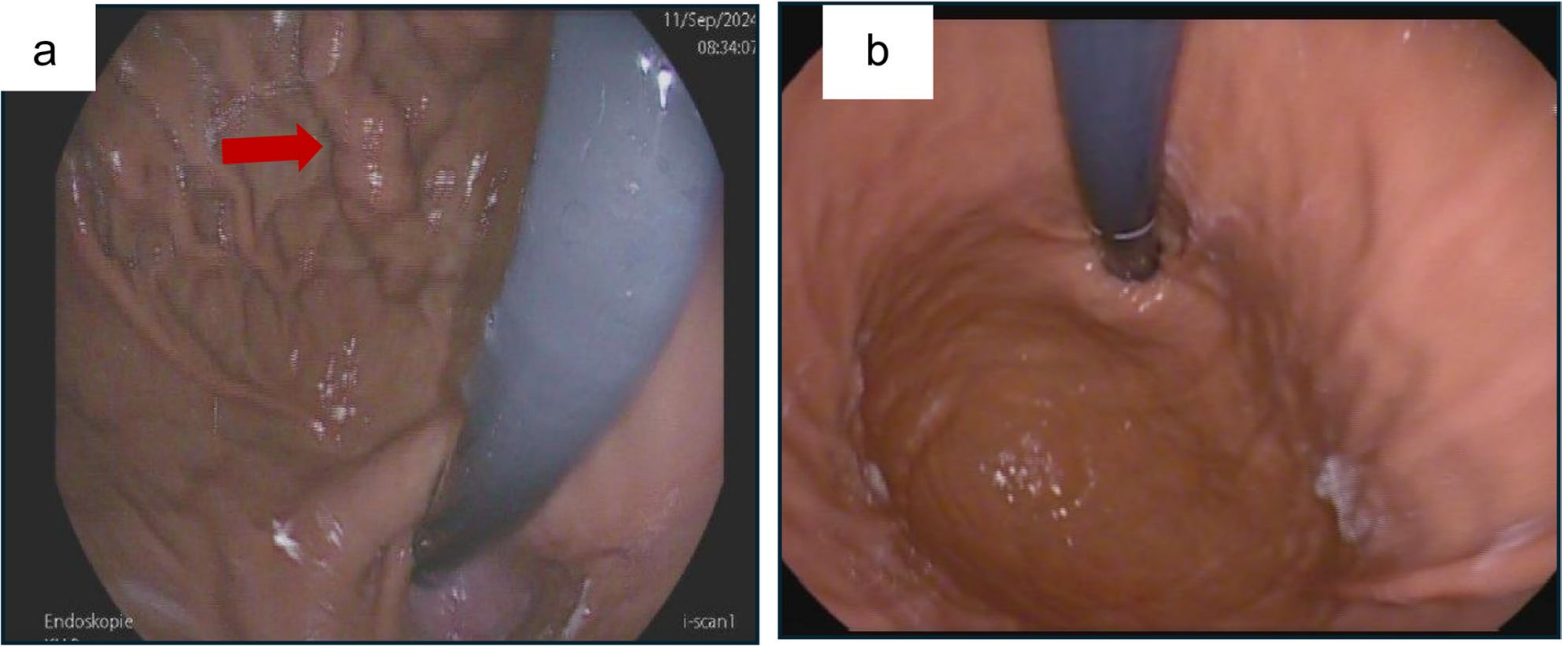
Esophagogastroduodenoscopy in a 71-year-old woman with idiopathic splenic vein stenosis presenting with persistent epigastric pain, (**a**) shows prominent gastric fundal varices (red arrow) before splenectomy. **b** shows complete resolution of the varices at 2 months post-splenectomy

**Fig. 2:**
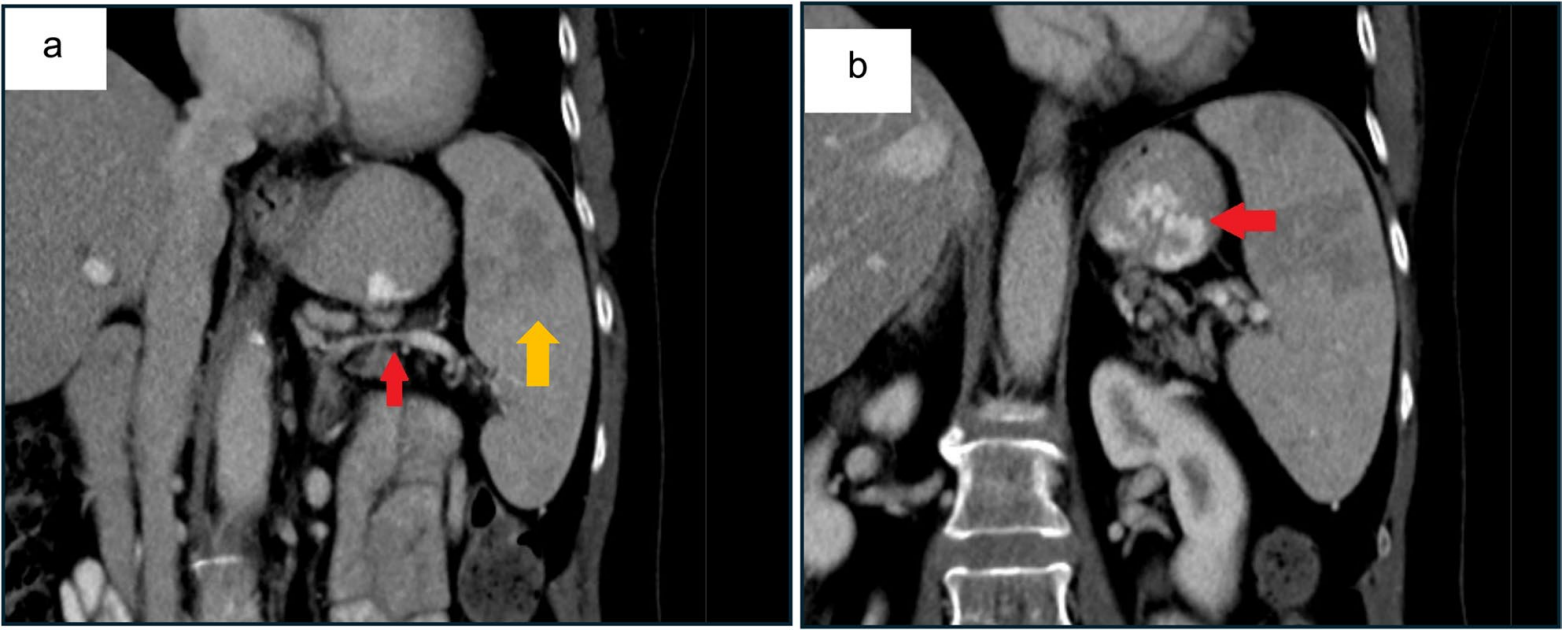
Coronal multiphasic CT in a 71-year-old woman with idiopathic splenic vein stenosis. **a** reveals the area of stenosis in the splenic vein (red arrow) and a large area of splenic infarction (2a, yellow arrow). **b** shows prominent venous collaterals at the pancreatic tail and posterior gastric fundal varix

**Fig. 3:**
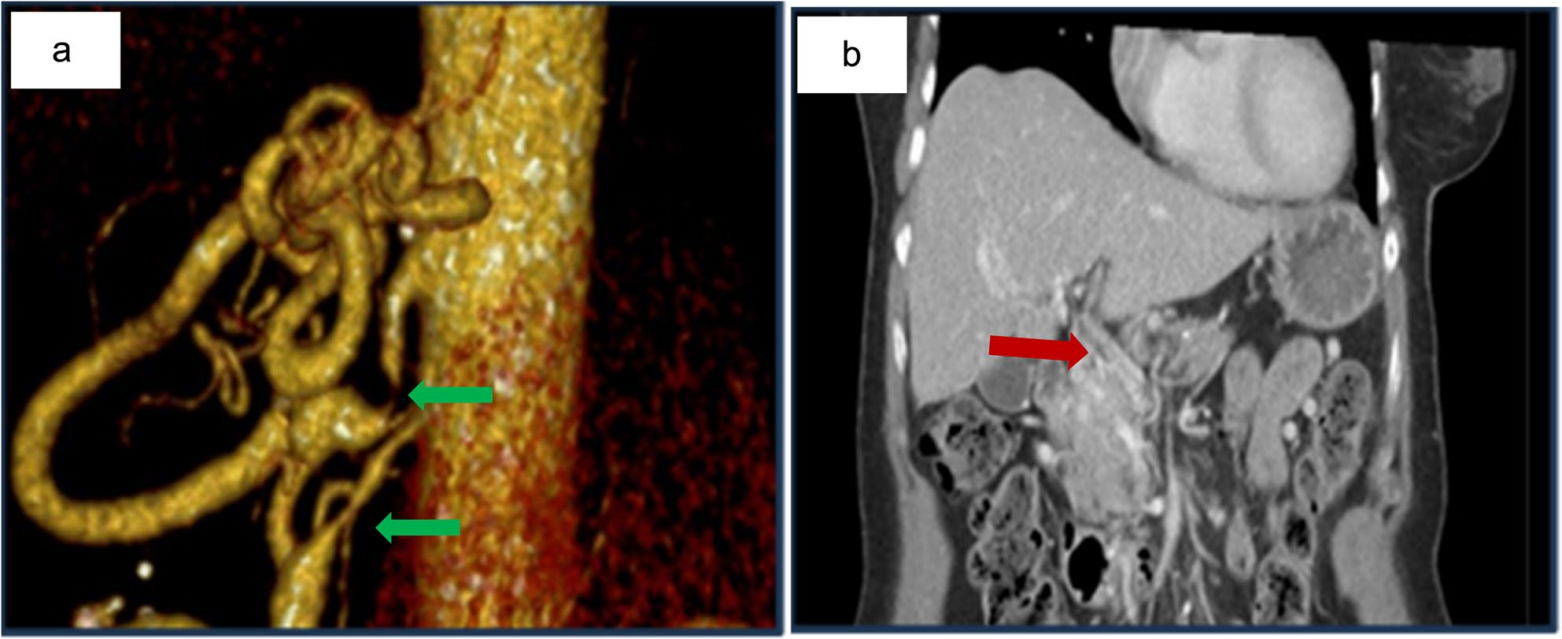
**a** shows a reconstructed CT image revealing high-grade stenoses of the celiac trunk and superior mesenteric artery (green arrows) with a dilated Riolan anastomosis and **b** shows portal vein thrombosis post-splenectomy in a 71-year-old woman with idiopathic splenic vein stenosis

Upper gastrointestinal endoscopy revealed large, tortuous type I gastric varices (Fig. 1a). Multiphasic contrast-enhanced CT demonstrated high-grade splenic vein stenosis as shown in Fig. 2a, with prominent venous collaterals at the pancreatic tail and posterior gastric fundal varices (Fig. 2b). A wedge-shaped perfusion deficit in the spleen, indicative of splenic infarction, and moderate splenomegaly was noted (Fig. 2a/b), potentially contributing to her discomfort. No pancreatic masses, pseudocysts, or inflammatory changes were identified, and there were no signs of intrabdominal malignancy or inflammatory changes such as retroperitoneal Fibrosis. Incidentally, CT revealed high-grade stenoses of the celiac trunk and superior mesenteric artery (SMA) with a dilated Riolan anastomosis (Fig. 3a).

These arterial stenoses were deemed hemodynamically insignificant and did not necessitate further intervention at the time. Following these imaging findings, a rheumatologic workup was performed, yielding negative results and effectively excluding systemic vasculitis (Table 1).

### Management and follow-up

**Fig. 4:**
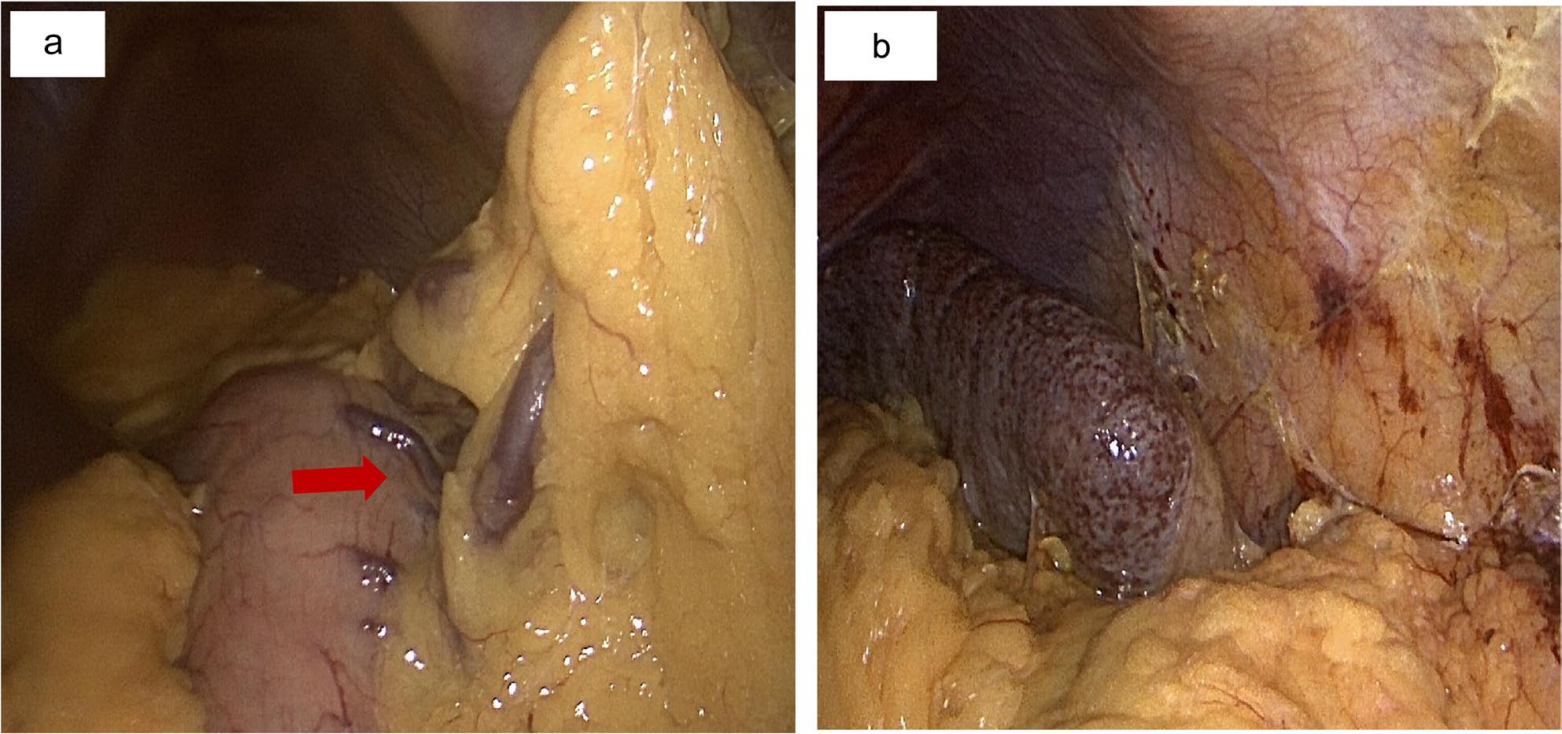
Laparoscopic images showing tortuous gastric varices (**a**) and hyperemic spleen from chronic venous congestion (**b**) in a 71-year-old woman with idiopathic splenic vein stenosis presenting with persistent epigastric pain, showing prominent gastric fundal varices (red arrow)

The presence of symptomatic splenic infarction and concern for variceal bleeding prompted evaluation for definitive surgical management. Following appropriate immunization with pneumococcal vaccine, the patient underwent an uncomplicated laparoscopic splenectomy. Intraoperative findings revealed extensive peri-splenic venous collaterals (Fig. 4a/b). Histopathological examination of the resected spleen showed no evidence of malignancy, vasculitis, or other underlying pathology. Her immediate postoperative recovery was uneventful, with complete resolution of epigastric discomfort. Postoperatively, prophylactic anticoagulation therapy was administered and discontinued at discharge after ruling out clinical indication for an extended anticoagulation therapy.

At routine follow-up two months after surgery, doppler ultrasound of the abdomen demonstrated reduced portal vein patency and an intraluminal thrombus. Subsequent contrast-enhanced CT angiography confirmed cavernous transformation of the portal vein, consistent with newly developed portal vein thrombosis (Fig. 3a). There was no radiological evidence of intra-abdominal malignancy or recurrent varices.

In the absence of clinical or laboratory features suggestive of systemic vasculitis and with no apparent prothrombotic risk factors on initial evaluation, the patient was referred to hematology for further workup to assess for inherited or acquired coagulopathy, with no positive findings (Table 2 showing key coagulation parameters in the supplement material). She was empirically started on oral anticoagulation therapy while awaiting further results.

A follow-up gastroscopy performed two, four and eight months postoperatively showed near-complete regression of the gastric fundal varices, with no evidence of esophageal varices (Fig. 1b). The patient remains asymptomatic and continues anticoagulation therapy, with plans for ongoing serial imaging and endoscopic surveillance.

## Discussion

### Case novelty and clinical significance

This report presents an uncommon manifestation of idiopathic splenic vein stenosis (SVS), marked by persistent epigastric discomfort in the absence of gastrointestinal bleeding, subsequently complicated by splenic infarction and post-splenectomy portal vein thrombosis (PVT) with cavernous transformation. To our knowledge, this represents the first reported case to describe such a continuum, thereby expanding the recognized clinical and vascular spectrum of this rare splanchnic disorder.

### Diagnostic approach to idiopathic splenic vein stenosis

The diagnosis of idiopathic SVS in this patient was substantiated by imaging findings of high-grade splenic vein stenosis, isolated type I gastric fundal varices, normal liver biochemistry, a patent portal vein, and the absence of pancreatic, traumatic, or inflammatory pathology. These features are consistent with previously defined criteria for idiopathic etiology [1, 2, 5]. Unlike secondary SVS, typically linked to pancreatic disease, trauma, or malignancy, idiopathic SVS remains a diagnosis of exclusion. The current literature on idiopathic SVS is limited to case reports, most of which describe patients presenting with upper gastrointestinal bleeding [1, 3, 4]. Typical sequelae include isolated gastric fundal varices, splenomegaly, and occasionally hypersplenism.

### Concurrent arterial and venous pathology: Evidence for systemic splanchnic vasculopathy

The pathogenesis of idiopathic SVS remains incompletely understood. Proposed mechanisms include congenital venous malformations, idiopathic intimal fibrosis, or microvascular remodeling [1]. However, none of these adequately explain the concurrent arterial abnormalities observed in this case. High-grade stenoses of the celiac trunk and superior mesenteric artery (SMA), with compensatory dilation of the Riolan anastomosis, suggest the presence of a broader splanchnic vasculopathy involving both arterial inflow and venous drainage. This constellation may promote venous stasis, regional ischemia, and thrombotic risk. The absence of systemic vasculitis or malignancy on serology and histology supports a non-inflammatory, likely structural vascular etiology.

Splenic infarction, while commonly encountered in pancreatitis or traumatic settings, is rarely emphasized in idiopathic SVS and often remains an incidental radiologic finding [12, 13]. In our patient, the infarct was likely precipitated by chronic venous congestion and segmental perfusion impairment. The absence of systemic symptoms and normal inflammatory markers demonstrates the often-subclinical nature of such infarcts, and highlights the limitations of laboratory evaluation. In this context, contrast-enhanced cross-sectional imaging remains central to diagnosis and staging.

### Post-splenectomy portal vein thrombosis

Splenectomy remains the standard intervention for symptomatic SVS, particularly in cases of variceal bleeding, and in our case symptomatic splenic infarction [9, 10]. It offers effective decompression of the left-sided portal system and is associated with rapid variceal regression in patients with preserved liver function [1, 4]. Nonetheless, this case highlights a potentially underrecognized risk of post-splenectomy portal venous complications. Within two months of surgery, the patient developed PVT with cavernous transformation, an outcome not previously documented in cases of idiopathic SVS [1, 3, 4].

The pathophysiological mechanisms underlying post-splenectomy PVT are likely multifactorial. Sudden shifts in portal hemodynamics, manipulation of vascular endothelium during surgery, transient perioperative hypercoagulability, and pre-existing mesenteric arterial stenosis may collectively predispose to thrombosis. While routine coagulation studies were unremarkable, the possibility of undetected inherited thrombophilia or endothelial dysfunction cannot be excluded.

### Risk stratification and thromboprophylaxis strategies: Implication for clinical practice

This case challenges the conventional understanding of idiopathic SVS as a stable, segmental lesion. Rather, it highlights its potential to evolve into more diffuse portal hypertension in the postoperative period. It also highlights a crucial gap in surveillance protocols: although splenectomy is often considered curative, our findings support the need for extended post-operative monitoring including early doppler ultrasound or CT venography to detect evolving thrombotic complications before cavernous transformation becomes irreversible.

Furthermore, preoperative vascular evaluation should not be limited to the splenic vein but should routinely include arterial imaging. Identifying coexistent mesenteric arterial stenoses may guide perioperative risk stratification and influence the decision to initiate thromboprophylaxis [11, 14]. The role of prophylactic anticoagulation in the context of idiopathic SVS remains undefined, but current consensus guidelines recommend doppler ultrasound surveillance within the first postoperative week and extended thromboprophylaxis in at high risk patients including patient with haemato-oncological disorders [15–18].

### Alternative management strategies beyond splenectomy

This case also raises broader questions regarding the optimal management of SVS. While splenectomy is effective in many patients, emerging interventional strategies such as splenic vein stenting or selective embolization may offer less invasive alternatives in anatomically suitable cases [19, 20]. Future studies are needed to assess their safety and efficacy. In parallel, advances in vascular imaging (e.g., MR venography, contrast-enhanced ultrasound) and molecular diagnostics (e.g., endothelial gene panels) may aid in identifying structural or hereditary predispositions.

## Conclusion

This case expands the clinical phenotype of idiopathic SVS by documenting splenic infarction and post-splenectomy PVT with cavernous transformation. It underscores the need for comprehensive vascular assessment, including both arterial and venous systems, and calls for a reevaluation of perioperative management and surveillance in patients undergoing splenectomy for this rare condition. A heightened index of suspicion, coupled with individualized imaging and anticoagulation strategies, may help prevent overlooked complications and improve long-term outcomes.

## Supplementary Information

Supplementary Material 1.

## Data Availability

The datasets used and/or analyzed during the current study are available from the corresponding author on reasonable request **.**.

## Abbreviations

CT: Computer tomography
MRI: Magnetic resonance Imaging
PVT: Portal vein thrombosis
SMA: Superior mesenteric artery
SVS: Splenic vein stenosis
