# Supplementary material for: Idiopathic splenic vein stenosis with splenic infarction: a case report of rare non-bleeding cause of left-sided portal hypertension
Source: BMC Gastroenterol. 2026 Apr 10;26:228. doi: 10.1186/s12876-026-04783-9 (PMC13072485; doi:10.1186/s12876-026-04783-9)
Supplement: Supplementary file 1 — Supplementary Material 1. [file 12876_2026_4783_MOESM1_ESM.docx]

Table 1.: Laboratory results of a patient with idiopathic splenic vein stenosis presenting with isolated gastric varices and splenic infarction summarizing the key hematological, biochemical, rheumatological findings of a patient diagnosed with idiopathic splenic vein stenosis, who presented with isolated gastric varices and evidence of splenic infarction.

| **Serum biochemical** | **Unit** | **Patient value** | **Reference** | **Rheumatological Parameter** | **Units** | **Patient Value** | **Reference** |
| --- | --- | --- | --- | --- | --- | --- | --- |
| CRP | mg/l | 0.6 | < 5.0 mg/l | IgA | mg/dl | 265 | 70-500 |
| TSH basal | mU/l |  | 0.38 – 5.33 | IgG | mg/dl | 1107 | 700-1600 |
| GOT (ASAT) | μmol/l·s | 0.38 | < 0.60 | IgM | mg/dl | 402 | 40- 280 |
| GPT (ALAT) | μmol/l·s | 0.32 | < 0.60 | Immunix |  | Negative | Negative |
| GGT | μmol/l·s | 0.30 | < 0.63 | Dsdns | IU/ ml | >10 | <100 |
| Lipase | μkat/l | 0.43 | 0.22 – 1.0 | NukleoAK | Negativ | Negative | Negative |
| LDH | μmol/l·s | 2.62 | < 4.12 | SMAK | Negativ | Negative | Negative |
| Albumin | μmol/l | 45.6 | 35 – 52 | POAK | Negativ | Negative | Negative |
| Bilirubin | μmol/l | 7 | < 21 | PCNAAK | Negativ | Negative | Negative |
| Creatinine | μmol/l | 69 | 53 – 88 | HISTAK | Negativ | Negative | Negative |
| eGFR (CKD-EPI) | ml/min/1.73 | 77 | ≥ 90 | U1SNRNP | Negativ | Negative | Negative |
| Urea | mmol/l | 4.0 | < 11.9 | ROSSA52 | Negativ | Negative | Negative |
|  |  |  |  | ROSSA60 | Negativ | Negative | Negative |
| **Hematology parameters** |  | **Patient value** | **Reference** | LASSBAK | Negativ | Negative | Negative |
| Erythrocyte (RBC) | T/l | 4.90 | 3.85 – 5.20 | SCL70AK | Negativ | Negative | Negative |
| Haemoglobin | mmol/l | 8.8 | 7.3 – 9.8 | CENPBAK | Negativ | Negative | Negative |
| Haematocrit | l/l | 0.44 | 0.35 – 0.46 | JO1AK | Negativ | Negative | Negative |
| MCV | fl | 90 | 80 – 101 | MI2AK | Negativ | Negative | Negative |
| MCH | fmol | 1.79 | 1.67 – 2.11 | PMSCLAK2 | Negativ | Negative | Negative |
| MCHC | mmol/l | 20.0 | 19.6 – 22.4 | KUAK | Negativ | Negative | Negative |
| RDW-SD | fl | 44.6 | 39 – 51 | ANA17G | Negativ | Negative | Negative |
| RDW-CV | % | 14.1 | 11.5 – 15.5 | c-ancaT |  | Titer < 1:10 | Titer < 1:10 |
| Platelet Count | G/l | 138 | 160 – 370 | p-ancaT |  | Titer < 1:10 | Titer < 1:10 |
| Leukocyte | G/l | 8.5 |  | Rf | IU/ ml | <20.0 | <20 |
|  |  |  |  |  |  |  |  |
| **Differential leukocyte count** |  | **Percentage (Reference)** | **Absolute (G/l) (Reference)** | **Anemia Diagnostics** |  | **Patient Value** | **Reference** |
| Neutrophils | % | 66 (42 – 77) | 5.64 (1.50 – 7.70) | sTRF | mg/l | 0.85 | 0.9 – 2.01 |
| Lymphocytes | % | 26 (20 – 44) | 2.18 (1.10 – 4.00) | Ferritin | ng/ml | 115 | 13-140 |
| Monocytes | % | 6.1(2.0 – 9.5) | 0.52 (0.10 – 0.90) | EPO | IU/l | 5.7 | 3.7 – 29.5 |
| Eosinophils | % | 1.7 (0.5 – 5.5) | 0.15 (0.01 – 0.40) | Ana |  | >1:80 | < 1:80 |
| Basophils | % | 0.4 (0 – 1.8) | 0.03 (0 – 0.20) |  |  |  |  |
| ***IgA****: Immunoglobulin A,* ***IgG****: Immunoglobulin G,* ***IgM****: Immunoglobulin M,* ***Immunix,*** *Immunoblot Panel (Multiplex autoantibody test),* ***DSDNS****: Anti-dsDNA antibodies (Double-stranded DNA),* ***NukleoAK****: Anti-nucleosome antibodies,* ***SMAK****: Anti-smooth muscle antibodies (SMA),* ***POAK****: Anti-mitochondrial antibodies (AMA-M2),* ***PCNAAK****: Anti-PCNA antibodies (Proliferating Cell Nuclear Antigen),* ***HISTAK*** *: Anti-histone antibodies, ,* ***U1SNRNP:*** *Anti-U1 small nuclear ribonucleoprotein antibodies,* ***ROSSA52****: Anti-Ro/SSA 52 kDa antibodies,* ***ROSSA60****: Anti-Ro/SSA 60 kDa antibodies,* ***LASSBAK****: Anti-La/SSB antibodies,* ***SCL70AK*** *: Anti-Scl-70 antibodies (Topoisomerase I),* ***CENPBAK****: Anti-centromere protein B antibodies* ***JO1AK*** *: Anti-Jo-1 antibodies (tRNA synthetase),* ***MI2AK*** *Anti-Mi-2 antibodies (helicase; seen in dermatomyositis),* ***PMSCLAK2****: Anti-PM/Scl antibodies (Type 2),* ***KUAK****: Anti-Ku antibodies,* ***ANA17G****: Antinuclear antibodies (17-antigen IgG immunoblot),* ***c-ANCA T:*** *Cytoplasmic ANCA Titer (Anti-PR3),* ***p-ANCA T*** *Perinuclear ANCA Titer (Anti-MPO)* ***RF****: Rheumatoid Factor,* ***sTRF:*** *soluble transferrin receptor;* ***Ferritin*** *:iron storage protein;* ***EPO:****erythropoietin;* ***ANA:*** *antinuclear antibodies.* | | | | | | | |

Table 2.: Laboratory results of a patient with idiopathic splenic vein stenosis presenting with isolated gastric varices and splenic infarction summarizing the key coagulation parameters in a patient diagnosed with idiopathic splenic vein stenosis, who presented with isolated gastric varices and evidence of splenic infarction.

| **Coagulation Parameter*** | **Unit** | **Patient value** | **Reference** | **Coagulation Parameter*** | **Unit** | **Patient Value** | **Reference** |
| --- | --- | --- | --- | --- | --- | --- | --- |
| Cardiolipin Antibody Type IgM | U/ml | 4.3 | 0.01 - 20 | Quick (Thromboplastin Time) | % | 40 | 70 - 150 |
| PAI-1 Antigen | ng/ml | 36.84 | 0.9 - 28.5 | INR | - | 1.9 | 0.01 - 3 |
| PAI-1 Activity | ng/ml | 1.00 | 0.01 - 5.1 | aPTT | sec | 37.4 | 25.3 - 37 |
| Von Willebrand Factor Antigen | % | 196.8 | 66 - 177 | Fibrinogen (Clauss) | mg/dl | 369 | 200 - 400 |
| Von Willebrand Ristocetin Cofactor | % | 143.7 | 60 - 241 | D-Dimers | ng/ml | 154 | < 500 |
| Coagulation Factor II | % | 65 | 70 - 130 | Fibrin Degradation Products | µg/ml | 0.3 | 0.01 - 2.02 |
| Coagulation Factor V | % | 80 | 60 - 140 | C-reactive Protein | mg/l | 3.3 | 0.01 - 5.1 |
| Coagulation Factor VII | % | 37 | 50 - 130 | APC Resistance | TR | 4.5 | Test Ratio |
| Coagulation Factor VIII | % | 94 | 50 - 150 | Factor V Leiden Mutation* | - | Wildtype | - |
| Coagulation Factor IX | % | 56 | 65 - 149 | Prothrombin Mutation G20210A* | - | Wildtype | - |
| Coagulation Factor X | % | 75 | 77 - 149 | Protein C Activity (chrom.) | % | 117 | 69 - 141 |
| Coagulation Factor XI | % | 64 | 65 - 149 | Protein C Activity (clot) | % | 158 | 69 - 141 |
| Coagulation Factor XII | % | 77 | 70 - 149 | Protein S Activity | % | 188 | 65 - 200 |
| Coagulation Factor XIII | % | 118 | 70 - 159 | Free Protein S | % | 67.7 | 54 - 200 |
| Anti-Xa Activity (Rivaroxaban) | ng/ml | 99.00 | - | Lupus Anticoagulant Screen | Ratio | 1.86 | < 1.20 |
| Antithrombin (Xa-based) | % | 117 | 82 - 130 | Lupus Ratio (LA1/LA2) | TR | 1.44 | 0.01 - 1.2 |
| Antithrombin (IIa-based) | % | 90 | 79 - 121 | Lupus Ratio Plasma Switch | TR | 1.21 | 0.01 - 1.2 |
| B2-Glycoprotein Antibody Type IgM | U/ml | 1.1 | 0.01 - 20 | Clotting Time Ratio (SCT1/SCT2) | TR | 0.95 | 0.01 - 1.2 |
| Cardiolipin Antibody Type IgG | U/ml | 8.0 | 0.01 - 20 | B2-Glycoprotein Antibody Type IgG | U/ml | 19.2 | 0.01 - 20 |
| ***PAI****: Plasminogen Activator Inhibitor,* ***IgG****: Immunoglobulin G, IgM: Immunoglobulin M,* ***aPTT*** : Activated Partial Thromboplastin Time, ***INR:*** International Normalized Ratio, ***APC:*** *Activated Protein C* | | | | | | | |
